# Comparative Antiviral Efficacy of Generic Sofosbuvir versus Brand Name Sofosbuvir with Ribavirin for the Treatment of Hepatitis C

**DOI:** 10.1155/2018/9124604

**Published:** 2018-10-01

**Authors:** L. Tang, M. Kamat, A. Shukla, M. Vora, C. Kalal, S. Kottilil, S. Shah

**Affiliations:** ^1^Division of Clinical Care and Research, Institute of Human Virology, University of Maryland, Baltimore, MD, USA; ^2^Department of Hepatology, Institute of Liver Diseases, Hepato Pancreato Biliary Surgery and Transplant, Global Hospitals, Mumbai, India

## Abstract

**Background:**

Chronic hepatitis C infection is a major cause for liver failure and liver cancer and can be treated with highly effective all oral directly acting antiviral (DAA) drugs. Generic versions of these DAAs are available in India.

**Method:**

This was an open-label, single-center, prospective, nonrandomized observational study for the comparative safety and efficacy of generic versus brand name sofosbuvir with ribavirin therapy for chronic hepatitis C infection (all genotypes). Between December 2014 and December 2015, 66 patients received either generic sofosbuvir (400mg) or brand name SOLVALDI (400mg) with weight based ribavirin for 24 weeks in a single multispecialty hospital in Mumbai. Monitoring viral loads and safety labs was performed as per national guidelines.

**Results:**

Sustained virologic response was 72.4% versus 75.7% (*p*=0.78) among patients treated with generics and SOVALDI, respectively. At 4 weeks on-treatment, approximately 90% of patients from both groups had undetectable or below the lower limit of quantification. Change in hemoglobin was comparable in both groups (p=0.26).

**Conclusion:**

Generic versions of sofosbuvir reported in this study are safe and efficacious to treat hepatitis C. However, bioequivalency studies of all generic DAAs need to be performed before wider use of such drugs for the treatment of hepatitis C.

## 1. Introduction

Approximately 700,000 persons worldwide die each year from liver disease related to chronic hepatitis C viral (HCV) infection [1]. Chronic HCV continues to be the leading cause of cirrhosis, liver failure, hepatocellular carcinoma and death globally [2, 3]. Global prevalence of HCV is estimated at 1%, which means that approximately 71.1 million people are infected [4]. The prevalence of HCV in India is estimated to range from 0.09% to 2.02% [5, 6]. While this falls in the low to moderate range, when the population size of India (over 1.2 billion) is considered, it is likely that approximately 20 million people may be living with HCV in India, which is a significant proportion of the global burden of disease.

Highly effective direct acting antiviral (DAA) drugs for chronic HCV infection are now available, leading many to discuss the possibility of eliminating HCV. However, development of curative therapy is only one of many steps required for elimination. Access to qualified care and the medications themselves are also vital steps, and the high price of these medications has severely limited access, especially in low and middle income countries such as India [7]. Recently, generic DAA's are now being manufactured in several Asian countries and generic sofosbuvir is made available at 1% of the cost of SOVALDI [8]. Recently, several observational reports on the sustained virologic response rates (SVR, considered cure) of over 95% with generic DAA drugs (sofosbuvir, ledipasvir, daclatasvir, and velpatasvir) have been published [9–14], including among patients with chronic kidney disease [13], and patients with HIV coinfection [15]. However, few studies have been performed in India. Furthermore, few included a high proportion of patients with genotype 3 infection, which is more prevalent in India [16, 17].

At the Institute of Liver Diseases, Hepato Pancreato Biliary Surgery and Transplant, Global Hospitals, Mumbai, India, SOVALDI became available in mid-2014. Generic sofosbuvir followed almost one year later. In this study, we report the real-world experience of safety, tolerability, and SVR rates of HCV treatment with generic sofosbuvir and compare this to brand name sofosbuvir (SOVALDI) in combination with ribavirin at this site.

## 2. Materials and Methods

### 2.1. Patients

From December 2014 to December 2015, consecutive patients were treated with sofosbuvir and ribavirin at the Institute of Liver Diseases, Hepato Pancreato Biliary Surgery and Transplant, Global Hospitals, Mumbai, India. Both interferon/ribavirin treatment-experienced, and treatment-naïve patients of any fibrosis staging were eligible for evaluation for treatment. All patients who initiated treatment were prospectively observed and outcomes analyzed.

### 2.2. Fibrosis Staging

The presence or absence of cirrhosis and differentiation between decompensated and compensated cirrhosis was determined by the treating clinician. Cirrhosis was diagnosed based on local clinical criteria that consisted of any combination of the following: the presence of cirrhosis on radiologic scan, documented diagnosis of esophageal varices or collaterals, and/or peripheral stigmata of cirrhosis on physical exam.

### 2.3. Treatment

Patients were treated per standard of care. Patients received either brand name sofosbuvir (SOVALDI) or generic sofosbuvir (Table 1) in combination with generic ribavirin. The decision as to which sofosobuvir to utilize, dosing of ribavirin, and duration of therapy was made by the prescribing clinician. Ribavirin dose was based on weight (1000 mg for weight <75 kg and 1200 mg for weight >75 kg). Adherence to treatment was monitored with patient reports.

Monitoring HCV viral loads and safety labs (complete blood, renal function, and liver enzymes) were performed as per national guidelines. Adverse events recorded included hemoglobin decline, blood transfusions required, death, and transplantation. Hemoglobin decline was graded according to the National Institute of Allergy and Infectious Diseases (NIAID) Division of AIDS (DAIDS) grading of adult adverse events (version 2.1) where grade 3 events are considered severe, and grade 4 are considered potentially life-threatening. The number of patients who did not complete treatment and the reasons for noncompletion was also collected.

### 2.4. Virologic Response

Sustained virologic response (SVR) was defined as undetectable HCV RNA levels measured at least 12 weeks after completion of DAA therapy and represents cure. HCV RNA was measured by locally available assays using real-time PCR with the lower limit of detection at 34 IU/ml.

### 2.5. Statistical Analysis

Baseline characteristics and rates of SVR between patients treated with generic sofosbuvir and SOVALDI were compared. Differences in mean and observed frequency were analyzed with the t-test and Fisher's exact test, respectively. A two-sided p value less than 0.05 was considered significant.

### 2.6. Institutional Approval

This study was approved by the institutional ethics committee of Global Hospitals, Mumbai, India.

## 3. Results

### 3.1. Baseline Characteristics

Sixty-six patients were started on treatment during the observed period. Twenty-nine patients received generic sofosbuvir and 37 received SOVALDI in combination with ribavirin. Overall, baseline characteristics between treatment groups did not differ significantly (Table 1). All patients were of Indian origin, with an overall mean age of 54.3 years. Numbers of males and females were essentially equal. The majority of patients were cirrhotic (N=45, 68% of all patients, 20 [69%] in the generic group, and 25 [67.6%] in the SOVALDI group), with approximately half of cirrhotics being decompensated (N=20). There were marginally more decompensated patients in the SOVALDI group compared to the generic group (15 out of 25 compared to 5 out of 20 patients,* p*=0.034). At the treatment center, SOVALDI was available prior to generic formulations. Patients with advanced liver disease were among the first to be initiated on treatment once SOVALDI became available. This may explain the marginally higher number of patients with decompensated cirrhosis in the SOVALDI group compared to the generic group. Baseline Child-Pugh and Model for End-Stage Liver Disease score (MELD score, a predictor of 3 month mortality risk among patients with end-stage liver disease [18], Table 1) scores were similar in both groups. Infection with HCV genotype 3 predominated. The proportion of patients with genotype 3 infection was higher in the generic group compared to the SOVALDI group (26 out of 29, 89.7% compared to 23 out of 37, 62.6%,* p*=0.013). Most were naïve to any HCV treatment, with only 13 (20%) having experience with interferon-ribavirin therapy. Table 1 details the baseline characteristics for patients who initiated treatment.

### 3.2. Virologic Response

**Figure 1:**
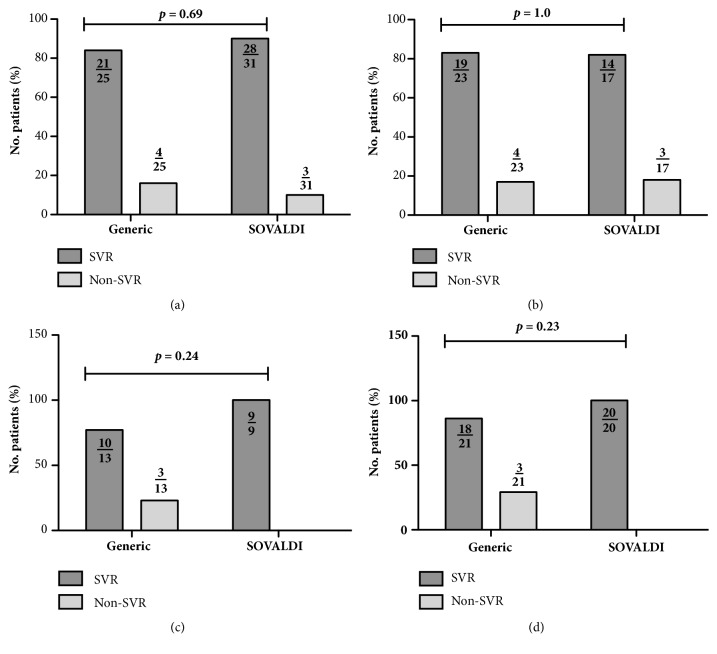
Virologic response among patients with available sustained virologic response (SVR). (a) Percentage of all patients. (b) Percentage of genotype 3 patients. (c) Percentage of all patients with compensated cirrhosis. (d) Percentage of all patients excluding decompensated cirrhosis.

Table 1 summarizes the overall viral responses. At 4 weeks on treatment, 26 (89.7%) of all patients treated with generic sofosbuvir had undetectable or below lower limit of quantification viral loads, compared to 34 (91.9%) treated with SOVALDI (*p*=1). SVR data was missing for 4 patients who received generic sofosbuvir, and 6 who received SOVALDI. Of the patients with SVR data available, 21 (84%) from the generic sofosbuvir group and 28 (90.3%) from the SOVALDI group obtained SVR, while 4 (16%) and 3 (9.7%) did not (Figure 1). There was no statistically significant difference in SVR between the generic and SOVALDI group (*p*=0.69) including among the treatment experienced patients (Table 2). After excluding patients with decompensated cirrhosis, SVR was 75% (18/24 of all patients) and 91% (20/22 of all patients,* p*=0.25) among the generic and SOVALDI groups, respectively (Table 2).

Among all patients treated with generic sofosbuvir, 4 (13.8% of all patients, 16% of patients with available SVR data) virologic failures were observed. Three (8.1% of all patients, 9.7% of all patients with available SVR data) failures were observed in the SOVALDI group. All were cirrhotic with genotype 3 infection, and all completed their prescribed duration of therapy. In the generic group, of the 4 were men, 2 were treatment experienced, and 1 was diagnosed with decompensated cirrhosis at baseline with a mean baseline MELD of 9 (range 7 to 11). In the SOVALDI group, 2 were men, all were treatment naïve, and all were decompensated cirrhotics. Mean baseline MELD in the SOVALDI failure group was 14 (range 13 to 16).

### 3.3. Cirrhotics

Twenty patients with cirrhosis were initiated on dual therapy with generic sofosbuvir and ribavirin. SVR data is absent from 3 patients from this group: one died prior to SVR timepoint, and the other 2 were lost to follow up. SVR was 65% of all patients initiated on treatment (13 out of 20) and 76.5% of patients with available SVR (13 out of 17). Mean MELD among cirrhotics who achieved SVR was 8.7 (range 6 to 13) and 9.7 (range 7 to 16) at baseline and end of treatment, respectively.

Among the 15 patients with compensated cirrhosis treated with generic sofosbuvir, 13 had SVR data available. Ten achieved SVR, and 3 virologic failures were observed. In comparison, among the 10 compensated cirrhotic patients treated with SOVALDI, 9 achieved SVR. The differences in SVR rates were not statistically significant (Table 2, Figure 1). Change in MELD scores among the patients with compensated cirrhosis was similar between treatment groups (generic +1.13 ± 2.2; SOVALDI +0.6 ± 0.97;* p*=0.48).

### 3.4. Genotypes 1 and 3

Only 2 patients with genotype 1 infection were treated with generic sofosbuvir, and 12 were treated with SOVALDI. SVR was confirmed in 1 of the 2 patients treated with generics, the other patient died of liver failure prior to the SVR time point. All 12 treated with SOVALDI achieved SVR.

Of the 26 patients with genotype 3 infection treated with generics, 19 achieved SVR. Twenty-three patients with genotype 3 infection were treated with SOVALDI. Fourteen achieved SVR. SVR rates were similar between the treatment groups (Table 2, Figure 1).

### 3.5. Duration of Therapy

Twenty-seven patients from the generics group completed the course of treatment. Of the 2 that did not, one died after 136 days of treatment, and the other received a liver transplant after only 65 days of treatment. From the SOVALDI group, 33 patients completed treatment. Of the remaining 4, all discontinued treatment due to adverse reactions. Three discontinued after less than 60 days. All 3 had decompensated cirrhosis. The first patient had further decompensation of liver disease secondary to hepatocellular carcinoma, the second patient had severe anemia refractory to blood transfusions and ribavirin dose reduction, and the third patient had severe anemia secondary to ribavirin and was unable to receive blood transfusions due to allergic reactions. A fourth patient discontinued treatment at 133 days due to bleeding gums, at which point ribavirin was discontinued and daclatasvir added.

Patients in the generic group received more days of therapy compared to the SOVALDI (p=0.016), reflecting the higher proportion of patients with genotype 3 infection. However, among patients with SVR data available, the difference in duration was no longer significant (p=0.093).

### 3.6. Change in Model for End-Stage Liver Disease (MELD) Score

Baseline MELD score did not vary significantly between treatment groups, with the majority of patients having a score less than 10. MELD did not vary significantly from baseline to end of treatment in the both groups; however, it did increase by a mean of 1.24 in the generic group, which was statistically significant (*p*=0.039).

### 3.7. Safety

#### 3.7.1. Hemoglobin Decline and RBV Dose Adjustments

All patients experienced a decreased in hemoglobin during treatment. Mean change in hemoglobin was comparable in both groups (generics -2.9 ± 1.79 g/dL vs. SOVALDI -2.7 + 1.33 g/dL;* p*=0.65). Four patients in the generics group had grade 3 hemoglobin drops and one had grade 4. Ribavirin dose was reduced in 16 patients, and 3 required a blood transfusion (all patients with grade 3 or 4 hemoglobin drops). In the SOVALDI group, 5 had grades 3 and 7 had grade 4 decrease in hemoglobin. The incidence of grade 3 or 4 hemoglobin change was not significantly different between both groups (*p*=0.26).

#### 3.7.2. Deaths and Treatment Incompletion

In the generics group, 2 patients died before SVR results could be obtained. Two patients did not complete the prescribed course of treatment: one died after 19 weeks, and the other underwent liver transplantation at 9 weeks (this patient achieved SVR).

The proportion of patients completing treatment was similar in both groups (22/29 vs. 33/37;* p*=0.69). In the SOVALDI group, 4 patients did not complete the prescribed course of treatment. Three had decompensated cirrhosis, and treatment was terminated in 1 due to progression of liver disease and 2 due to refractory anemia. One patient experienced bleeding gums and in this patient, RBV was stopped, and daclatasvir started. Three patients died from liver-related disease. SVR was confirmed in one patient prior to death.

## 4. Discussion

In this study, we observed no statistically significant difference in SVR among patients receiving generic sofosbuvir and those receiving SOVALDI for chronic hepatitis C infection. Generic sofosbuvir was well tolerated and effective among patients with a high proportion of advanced disease, and genotype 3 disease.

Several observational studies reporting high SVR rates with generic formulations of DAA drugs have recently been published. In China, 187 patients with genotype 1b infection, with and without cirrhosis were treated for 8 to 12 weeks with generic sofosbuvir/ledipasvir. Sustained virologic response rates were 97% in all patient groups [9]. The use of generics to treat patients with genotype 4 infection was described from several studies from Egypt. Two observational studies from Egypt compared treatment with generic and brand name sofosbuvir and daclatasvir [10], and generic sofosbuvir/ledipasvir [11]. SVR rates were high (over 98%) in all patient groups in both studies. In another large observational study from Egypt, 18,378 patients were treated with generic sofosbuvir and daclatasvir. SVR was 95% in all groups; however there was no brand name group included for comparison [12]. Few studies have described the efficacy of dual therapy with sofosbuvir and ribavirin. Dual therapy with sofosbuvir and ribavirin is no longer included as one of the first line regimens in the American and European national treatment guidelines due to the availability of more effective regimens, including pangenotypic and ribavirin-free regimens (namely sofosbuvir combined with velapatasvir or daclatasvir, and glecaprevir/pibrentasvir) [19, 20]. However, in countries where infection with genotypes 2 and 3 are dominant, dual therapy with sofosbuvir and ribavirin remains an important regimen. The Asian-Pacific Association for the Study of the Liver (APASL) treatment guidelines still recommends this regimen for genotype 2 (12 weeks for treatment naïve, and 16 or 24 weeks for interferon and ribavirin experienced) and 3 (24 weeks for treatment naïve only) infection [21].

SVR among our generic cohort was comparable to those of clinical trials involving SOVALDI [22]. Published real-world reports of the efficacy of SOVALDI in treating genotype 3 infections with advanced liver disease are scarce, and even more so for generic formulations of sofosbuvir. The Hepatitis C Virus Therapeutic Registry and Research Network (HCV-TARGET) is an international, prospective observational study enrolling patients with chronic HCV infection and being treated with DAA therapy from academic and community centers in the United States, Canada, Germany, and Israel. Outcomes from patients with genotype 3 infection were recently reported. Here, after excluding patients who did not complete the prescribed course of treatment, and those lost to follow up, 178 patients treated with SOVALDI and ribavirin were included for analysis. Over half were cirrhotic (53.9%). Overall SVR was 60.1% and among cirrhotics SVR was less, at 55.3% for treatment naïve patients (21 out of 38) and only 37.9% (22 out of 58) for treatment experienced [23]. Our study is one of the first to report the positive real-world experience of transitioning from SOVALDI to generic sofosbuvir with ribavirin in the treatment of treatment naïve and pegylated interferon-ribavirin treatment experienced patients with predominantly genotype 3 infection, and cirrhosis. SVR appeared higher in our cohort of patients with cirrhosis compared to that reported in HCV-TARGET; however, the HCV-TARGET patients were predominantly white (82%) while our patients were all of Indian origin. Registrational studies of sofosbuvir in India recently reported high SVR rates among their patients with genotype 3 infection treated with sofosbuvir and ribavirin (over 90%) [24, 25]. Furthermore, an observational study of 490 patients treated with generic formulas of sofosbuvir combined with either ribavirin with or without pegylated interferon, or generic daclatasvir or ledipasvir, for 12 or 24 weeks at single center in India was recently published. Seventy-six percent of the patients had genotype 3 infection. Patients with cirrhosis were included, and 11.8% of patients had been previously treated with pegylated interferon and ribavirin. The overall SVR rate was 95.9% and among patients with genotype 3 infection, SVR rate was 96.5%. SVR was lower among treatment experienced patients compared to treatment naïve (87.9% versus 97%,* p*=0.005), a trend not observed in our cohort. Seventy-eight patients with genotype 3 infection were treated with sofosbuvir and ribavirin, and 77 (98.7%) achieved SVR. This study is one of the few to include the treatment of genotype 3 infection with generic formulations of sofosbuvir and ribavirin [14]. The SVR demonstrated in our study is more comparable to the published Indian studies and higher than US studies (TARGET). Therefore, ethnicity may affect efficacy.

Our study is limited by its non-randomized, observational design and small sample size. As such, it was not powered to detect non-inferiority to brand name sofosbuvir. The homogeneity in the ethnicity of all patients may be a further limitation in the generalizability of our findings. However, this is one of the earliest study to demonstrate safety and high SVR rates with generic sofosbuvir comparable to SVR rates with SOLVADI in a single center.

## 5. Conclusion

Treatment of chronic hepatitis C patients with generic sofosbuvir was well tolerated and demonstrated comparable SVR with those patients treated with SOLVADI in combination with ribavirin. This study is among the first to report on bioequivalency of new generics for HCV treatment. Future studies should focus on bioequivalency studies of all generics prior to wide spread use of generic DAAs to avoid safety concerns.

## Figures and Tables

**Table 1:** Patient characteristics.

Characteristic N (%)		Generic sofosbuvir		SOVALDI		P (generic versus SOVALDI)	
		All patients N=29	Patients with SVR data N=25	All patients N=37	Patients with SVR data N=31	All patients	Patients with SVR data
Male		13 (44.8)	12 (48)	17 (45.9)	13 (41.9)	1	0.79

Age (years) ± SD		56.86± 11.58	56.28± 12.12	52.22± 11.02	51.77± 10.79	0.1	0.2

Generic sofosbuvir							
Hepcivir		9 (31)	7 (28)				
Hepcinate		9 (31)	9 (36)				
Resof		2 (6.9)	0				
Viroclear		1 (3.4)	1 (4)				
Sofocure		7 (24.1)	7 (28)				
Myhep		1 (3.4)	1 (4)				

Fibrosis stage							
Non-cirrhotic		9 (31)	8 (32)	12 (32.4)	11 (35.5)	1	1
Cirrhosis							
Compensated		15 (51.7)	13 (52)	10 (27)	9 (29)		
Decompensated		5 (17.2)	4 (16)	15 (40.5)	11 (35.5)		

Treatment experienced (interferon/RBV)		5 (17.2)	5 (20)	8 (21.6)	5 (16.1)	0.78	0.73

Genotype							
1							
Unsubtyped		0	0	3 (8.1)	3 (9.7)		
1a		0	0	1 (2.7)	1 (3.2)		
1b		2 (6.9)	1 (4)	7 (18.9)	7 (22.6)		
1c		0	0	1 (2.7)	1 (3.2)		

3							
Unsubtyped		15 (51.7)	14 (56)	21 (56.8)	16 (51.6)		
3a		7 (24.1)	6 (24)	2 (5.4)	1 (3.2)		
3i		4 (13.8)	3 (12)	0	0		

4							
Unsubtyped		0	0	1 (2.7)	1 (3.2)		
4c		0	0	1 (2.7)	1 (3.2)		

6q		1 (3.4)	1 (4)	0	0		

Baseline CPS	Mean	5.72	5.48	6	5.9	0.39	0.21

EOT CPS	Mean	5.86	5.58	5.84	5.61	0.94	0.85

Baseline MELD*∗∗∗*	Mean	8.7	8.5	9.1	9	0.51	0.57

	<10	21 (72.4)	19 (76)	25 (67.6)	22 (71)		
	10-19	8 (27.6)	6 (24)	11 (29.7)	8 (25.8)		
	20-29	0	0	1 (2.7)	1 (3.2)		

EOT MELD	Mean	9.9	9.3	9.2	9	0.4	0.74

	<10	17 (58.6)	17 (68)	25 (67.6)	22 (71)		
	10-19	12 (41.4)	8 (32)	11 (29.7)	8 (25.8)		
	20-29	0	0	1 (2.7)	1 (3.2)		

Cirrhotics only	Mean baseline CPS	5.95	5.65	6.48	5.9	0.22	0.52

	Mean EOT CPS	6.1	5.77	6.24	5.61	0.75	0.65

	Mean baseline MELD	9	8.77	10.12	9	0.26	0.8

	Mean EOT MELD	10.65	10.12	10.32	9	0.75	0.25

Mean baseline HCV RNA (IU/ml) ± SD		3.07 x 10^6^ ± 6.69 x 10^6^	3.38 x 10^6^ ± 7.68 x 10^6^	1.02 x 10^7^ ± 2.38 x 10^7^	1.34 x 10^7^ ± 2.97 x 10^7^	0.72	0.13

Completed treatment		27	23	33	31	0.69	0.44

Mean baseline hemoglobin		12.83 ± 1.75	13.1 ± 1.73	12.44 ± 1.91	12.42 ± 1.76	0.39	0.19

Mean decrease in hemoglobin (g/dl) ± SD		2.78 ± 1.31	3.03 ± 1.81	2.99 ± 1.67	2.94 ± 1.35	0.62	0.86

Mean duration of therapy (days)*∗∗*		166.1 ± 22.13	165.8 ± 22.03	146.3 ± 38.3	155.2 ± 23.96	0.016	0.093

Sustained Virologic Response		21(72.4)	21 (84)	28 (75.6)	28 (90.3)	0.59	0.69

Treatment failure*∗*		8 (27.6)	4 (16)	9 (24.3)	3 (9.7)		

Week 4 on-treatment undetectable or below lower limit of detection		26 (89.7)	-* *-	34 (91.9)	-* *-	1	-* *-

*∗*Includes patients with missing sustained virologic response data (4 and 6 from the generic and SOVALDI cohorts respectively); *∗∗*one patient from the generic cohort discontinued treatment early (65 days), undergoing liver transplant at that time. This patient achieved sustained virologic response. *∗∗∗*Estimated 3-month mortality based on MELD scores [18]: 9 or less, 1.9%; 10-19, 6%; 20-29, 19.6%; 30-39, 52.6%; 40, 71.3%.

SVR: sustained virologic response; RBV: ribavirin; CPS: Child-Pugh score; EOT: end of treatment; MELD: Model for End Stage Liver Disease; HCV RNA: hepatitis C ribonucleic acid.

**Table 2:** Virologic response by patient subgroups.

Patient group	N (%)				P (generic versus SOVALDI)	
	Generic sofosbuvir		SOVALDI			
	All patients	Patients with SVR data	All patients	Patients with SVR data	All patients	Patients with SVR data
Overall SVR	N=29	N=25	N=37	N=31		
	21 (72.4)	21 (84)	28 (75.7)	28 (90.3)	0.78	0.69

Excluding decompensated cirrhotics	N=24	N=21	N=22	N=20		
	18 (75)	18 (85.7)	20 (90.9)	20 (100)	0.25	0.23

Genotype 3	N=26	N=23	N=23	N=17		
	19 (73.1)	19 (82.6)	14 (60.9)	14 (82.4)	0.54	1.0

Compensated cirrhotics	N=15	N=13	N=10	N=9		
	10 (66.7)	10 (76.9)	9 (90)	9 (100)	0.34	0.24

Treatment experienced	N=5	N=5	N=8	N=5	1.0	0.44
	3 (60)	3 (60)	5 (62.5)	5 (100)		

SVR: sustained virologic response.

## Data Availability

The data used to support the findings of this study are included within the article.
